# Prehospital management of patients with suspected acute coronary syndrome: Real world experience reflecting current guidelines

**DOI:** 10.1007/s00063-020-00739-3

**Published:** 2020-10-08

**Authors:** V.-S. Eckle, S. Lehmann, B. Drexler

**Affiliations:** 1 Medizinische Klinik mit Schwerpunkt Infektiologie und Pneumologie, Charité—Universitätsmedizin Berlin, Charitéplatz 1, 10117 Berlin, Germany; 2 Klinik für Anästhesiologie und Intensivmedizin, Universitätsklinikum Tübingen, Tübingen, Germany

**Keywords:** Time of prehospital care, Cardiac catheterization laboratory, Prehospital ECG, STEMI, NSTEMI, Präklinische Zeiten, Herzkatheter, Präklinisches Elektrokardiogramm, STEMI, NSTEMI

## Abstract

**Background:**

In case of suspected acute coronary syndrome (ACS), international guidelines recommend to obtain a 12-lead ECG as soon as possible after first medical contact, to administrate platelet aggregation inhibitors and antithrombins, and to transfer the patient as quickly as possible to an emergency department.

**Methods:**

A German emergency care service database was retrospectively analysed from 2014 to 2016. Data were tested for normal distribution and the Mann–Whitney test was used for statistical analysis. Results are presented as medians (IQR).

**Results:**

A total of 1424 patients with suspected ACS were included in the present analysis. A 12-lead ECG was documented in 96% of patients (*n* = 1369). The prehospital incidence of ST-segment elevation myocardial infarction (STEMI) was 18% (*n* = 250). In 981 patients (69%), acetylsalicylic acid (ASA), unfractionated heparin (UFH), or ASA and UFH was given. Time in prehospital care differed significantly between non-STEMI (NSTEMI) ACS (37 [IQR 30, 44] min) and STEMI patients (33 [IQR 26, 40] min, *n* = 1395, *p* < 0.0001). Most of NSTEMI ACS and STEMI patients were brought to the emergency care unit, while 30% of STEMI patients were directly handed over to a cardiac catheterization laboratory.

**Conclusions:**

Prehospital ECG helps to identify patients with STEMI, which occurs in 18% of suspected ACS. Patients without ST-elevations suffered from longer prehospital care times. Thus, it is tempting to speculate that ST-elevations in patients prompt prehospital medical teams to act more efficiently while the absence of ST-elevations even in patients with suspected ACS might cause unintended delays. Moreover, this analysis suggests the need for further efforts to make the cardiac catheterization laboratory the standard hand-over location for all STEMI patients.

## Abstract (de)

**Einführung:**

Internationale Leitlinien empfehlen die umgehende Ableitung eines 12-Kanal-Elektrokardiogramms bei Patienten mit akuter Angina-pectoris-Symptomatik. Ebenfalls wird die präklinische Gabe von Azetylsalizylsäure, Heparin sowie der sofortige Transport in ein Krankenhaus mit der Möglichkeit einer koronarinvasiven Diagnostik empfohlen.

**Methoden:**

Es wurden die Einsatzprotokolle einer Rettungsstellendatenbank retrospektiv für den Zeitraum von 2014–2016 auf diese Merkmale untersucht. Die Daten wurden auf Normalverteilung getestet und für die statistische Analyse wurde ein Mann-Whitney-Test verwendet. Die Ergebnisse sind als Mediane (Interquartilsabstand, IQR) dargestellt.

**Ergebnisse:**

Es wurden 1424 Patienten mit Angina-pectoris-Symptomatik eingeschlossen. In 96 % (*n* = 1369) der Fälle wurde ein 12-Kanal-Elektrokardiogramm aufgezeichnet. Präklinisch wurden 250 Patienten (18 %) mit ST-Strecken-Hebungs-Infarkt (STEMI) diagnostiziert. Insgesamt 981 Patienten (69 %) erhielten Azetylsalizylsäure bzw. Heparin oder Azetylsalizylsäure und Heparin. Die präklinischen Zeiten unterschieden sich signifikant zwischen Patienten mit akutem Koronarsyndrom ohne Ischämiezeichen im Elektrokardiogramm (NSTEMI ACS; 37 min [IQR 30,44]) und Patienten mit ST-Hebungen (STEMI; 33 min [IQR 26,40]). Der Großteil der Patienten wurde in der Notaufnahme übergeben, während 30 % der STEMI-Patienten direkt in das Herzkatheterlabor transferiert wurden.

**Schlussfolgerung:**

Die Inzidenz von ST-Hebungen liegt im präklinischen Bereich bei Patienten mit Angina-pectoris bei 18 %. Patienten ohne Ischämiezeichen wurden langsamer einem Krankenhaus zugeführt als Patienten mit STEMI. Dieses Ergebnis kann auf die geltenden Leitlinien zurückzuführen sein, da bei STEMI-Patienten die Zeit von Diagnosestellung bis zur koronarinvasiven Diagnostik <120 min betragen soll.

## Introduction

Atherosclerotic cardiovascular diseases are the leading cause of premature death in Europe with acute myocardial infarction being the second most common cause of death in Germany [13, 17]. One important measure for improving survival from an acute myocardial infarction is to reduce time delay from first medical contact to interventional coronary reperfusion therapy [8, 10]. Thus, prehospital electrocardiogram (ECG) recording by the emergency medical service after first medical contact in patients presenting with angina may facilitate prehospital triage management, enable an appropriate choice of destination hospital with cardiac catheterization laboratory and therefore reduce treatment delays [6, 7, 12]. Prehospital ECG is associated with lower mortality rates in patients presenting with acute myocardial infarction with ST-elevation (STEMI) or non-STEMI acute coronary syndrome (NSTEMI ACS) [14, 15]. International guidelines recognise prehospital ECG and recommend to obtain a 12-lead ECG as soon as possible after first medical contact in ACS patients [8, 10, 16].

In the present study, we investigated the impact of ST-elevations on time from first medical contact to arrival at the destination hospital, defined as time in prehospital care. Furthermore, we evaluated the handover location of STEMI and NSTEMI patients at the destination hospital.

## Methods

The retrospective study was approved by the University Hospital Tübingen ethics committee (approval reference number 401/2018BO1). The study region consisted of 225,000 inhabitants (Tübingen County, Germany). An emergency care service database (Deutsches Rotes Kreuz, Kreisverband Tübingen, Germany) of a mixed urban and rural area was retrospectively analysed from January 2014 to December 2016. Mainly, one local hospital with primary percutaneous coronary intervention facility (University Hospital Tübingen, Germany) served the study region. Local emergency care service cars were equipped with 12-lead ECG (Corpuls 3, GS Elektromedizinische Geräte G. Stemple GmbH, Kaufering, Germany).

All patients with the diagnosis of acute myocardial infarction with ST-elevation (STEMI) or non-STEMI acute coronary syndrome (NSTEMI ACS) were identified from emergency physician protocols (NADOK, Mindeststandard MIND 3.0/3.1 BW, DATAPEC GmbH, Pliezhausen, Germany) and included for further analysis. Exclusion criteria were missing data, no patient transportation or death of the patient before reaching the hospital. Time in prehospital care was defined as time from first medical contact to arrival at the destination hospital. Prehospital time points were obtained from the local emergency service (integrated emergency dispatch center Tübingen, Germany).

For quality reasons, these time points were double-checked with documentation from emergency physician protocols. In case of divergence of prehospital time points (deviation >10 min) by comparing both sources, emergency physician protocols were screened for obvious typos or documentation errors. If the error could not be identified and corrected, the respective time point was not used for further analysis. Measures and medication during time in prehospital care were investigated in accordance to current guidelines [10]. The data set was tested for Gaussian distribution by Kolmogorov–Smirnov–Lilliefors test (GraphPad Prism, GraphPad Software, San Diego, CA, USA) and the Mann–Whitney test was used for statistical analysis (*p* < 0.05). Results are presented as medians (IQR [interquartile range]).

## Results

In this retrospective study, 1442 patients with suspected acute coronary syndrome were analysed. A total of 18 data sets were excluded due to lack of data, no hospital referral or death so that 1424 patients were enrolled for further analysis. 1174 patients (82%) were prehospitally diagnosed with NSTEMI acute coronary syndrome (NSTEMI ACS) and 250 patients (18%) with STEMI.

After first medical contact, in 96% of patients (*n* = 1369) a prehospital 12-lead ECG was documented. In 96% of patients (*n* = 1367) an intravenous access was established. In 981 patients (69%) acetylsalicylic acid (ASA), unfractionated heparin (UFH), or ASA and UFH was given. From 573 patients presenting with pain (numeric rating scale ≥5) 456 patients (81%) received morphine as analgesic. Time on scene from first medical contact to transportation was 21 (IQR 16, 26) minutes (*n* = 1410).

Transportation time differed significantly in STEMI patients compared to patients with NSTEMI ACS (13 [IQR 8, 17] minutes vs. 15 [IQR 10, 20] minutes, *n* = 1401, *p* < 0.0001, Mann–Whitney test).

**Fig. 1:**
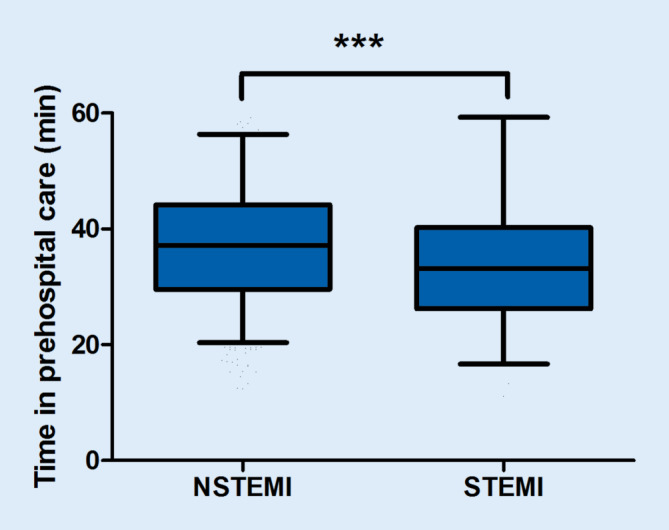
Time in prehospital care. *** *p* < 0.001. *NSTEMI* non-ST-segment elevation myocardial infarction, *STEMI* ST-segment elevation myocardial infarction

Time in prehospital care, defined as time from first medical contact to hospital admission was 37 (IQR 30, 44) minutes in NSTEMI ACS and 33 (IQR 26, 40) minutes in STEMI patients (*n* = 1395, *p* < 0.0001, Mann–Whitney test, Fig. 1).

In all, 89% of NSTEMI ACS patients (*n* = 1174) were transferred to an emergency care unit (Table 1). Most of STEMI patients were transferred to an emergency care unit (55%). Solely 30% of STEMI patients were handed over directly to a cardiac catheterization laboratory (Table 1).

**Table 1:** Patient handover locations at the destination hospital

	Emergency care unit	Emergency trauma room	Intensive care unit	Cardiac catheterization laboratory	Others	Total
*NSTEMI*	1049 (89)	6 (0.5)	13 (1)	7 (0.5)	99 (8)	1174
*STEMI*	137 (55)	3 (1)	16 (6)	75 (30)	19 (8)	250

Reported are patient numbers (%)

*NSTEMI* non-ST-segment elevation myocardial infarction, *STEMI* ST-segment elevation myocardial infarction

## Discussion

In the current study, we found an 18% incidence of STEMI in patients with acute coronary syndrome diagnosed by a prehospital ECG in a German district. In other studies, percentage of STEMI identification by prehospital ECG was 7% in Singapore, 38% in the United Kingdom, and 74% in Japan [9, 11, 14]. Our reported incidence is similar to a prospective cohort study from Sweden, where a prehospital ECG detected STEMI in 20% of patients with suspected acute coronary syndrome [19]. Also, a Dutch investigation showed a comparable incidence of 24% STEMI diagnosis by prehospital ECG in patients with acute chest pain [2].

Our main finding is that STEMI diagnosis leads to a shorter time in prehospital care compared to NSTEMI ACS patients. This may be explained—at least in part—by international guidelines as far as in case of STEMI time counts from ECG diagnosis, while for ACS patients without ST-elevations a time line is set up starting from first blood test for rule-in or rule-out NSTEMI [8, 10]. However, in patients with suspected NSTEMI high-sensitivity troponin should be analysed as soon as possible [1]. Thus, there should be no prehospital time delay in these patients.

In a Swedish prospective cohort study, prehospital ECG was associated with a reduced one-year mortality [15]. As far as 30% of STEMI patients were directly handed over to a catheterization laboratory, there should even recognized an effect on catheter lab team activation. However, the European Resuscitation Council Guidelines requires the readiness of the catheterization laboratory team within 20 min 24/7 [10]. This study reflects a large real world experience, non-biased by a Hawthorne effect [5]. Unfortunately, the catheterization laboratory is not the main handover location. This might have been caused at least in part by missing readiness of the laboratory team. Additionally, further efforts should be made, whether web-based transmission of prehospital ECG in case of STEMI ACS could increase the rate of catheter laboratory as handover location.

In this study population 69% of patients received aspirin (acetylsalicylic acid), heparin, or aspirin and heparin. In contrast, in an American database study, solely 45% of ACS patients received aspirin by an emergency medical service [18]. There are diverging recommendations regarding of administration of aspirin or anticoagulants in a prehospital setting for suspected NSTEMI ACS: While European Resuscitations Council guidelines recommend aspirin as soon as possible, the European Society of Cardiology points out that there is no data demonstrating a benefit for this practice [3, 4, 10]. From our data, it could not been derived whether patients not receiving aspirin from the emergency medical service had contraindications (e.g., an anaphylactic response) or had been taking aspirin before arrival of the emergency care team as primary or secondary prevention effort.

## Conclusions

The incidence of STEMI as diagnosed by prehospital ECG in patients with acute chest pain reaches 18% in a German district and is in accordance to epidemiologic data from Sweden and the Netherlands. In 96% of ACS patients, a prehospital 12-lead ECG was recorded. Prehospital ECG finding of ST-elevations has an impact on time in prehospital care so that STEMI patients arrived significantly earlier at the destination hospital. In fact, 30% of prehospital diagnosed STEMI patients were directly handed over to a coronary catheter laboratory. These data support the importance of ECG in prehospital care and management for patients suspected with acute coronary syndrome. Further research should focus on time from STEMI diagnosis to coronary reperfusion therapy including prehospital and clinical data.

## References

[1] Adler C, Baldus S (2019) Troponin elevation-does every patient require coronary angiography? Med Klin Intensivmed Notfmed (online ahead of print) 10.1007/s00063-019-0593-4 31218391

[2] Anroedh SS, Kardys I, Akkerhuis KM (2018). e-transmission of ECGs for expert consultation results in improved triage and treatment of patients with acute ischaemic chest pain by ambulance paramedics. Neth Heart J.

[3] Beygui F, Castren M, Brunetti ND (2015). Pre-hospital management of patients with chest pain and/or dyspnoea of cardiac origin. A position paper of the Acute Cardiovascular Care Association (ACCA) of the ESC. Eur Heart J Acute Cardiovasc Care.

[4] Beygui F, Castren M, Brunetti ND (2020). Pre-hospital management of patients with chest pain and/or dyspnoea of cardiac origin. A position paper of the Acute Cardiovascular Care Association (ACCA) of the ESC. Eur Heart J Acute Cardiovasc Care.

[5] Campbell JP, Maxey VA, Watson WA (1995). Hawthorne effect: implications for prehospital research. Ann Emerg Med.

[6] Carstensen S, Nelson GCI, Rasmussen HH (2007). Field triage to primary angioplasty combined with emergency department bypass reduces treatment delays and is associated with improved outcome. Eur Heart J.

[7] Chan AW, Kornder J, Elliott H (2012). Improved survival associated with pre-hospital triage strategy in a large regional ST-segment elevation myocardial infarction program. JACC Cardiovasc Interv.

[8] Ibanez B, James S, Agewall S (2018). 2017 ESC guidelines for the management of acute myocardial infarction in patients presenting with ST-segment elevation. Eur Heart J.

[9] Miyachi H, Takagi A, Miyauchi K (2016). Current characteristics and management of ST elevation and non-ST elevation myocardial infarction in the Tokyo metropolitan area: from the Tokyo CCU network registered cohort. Heart Vessels.

[10] Nikolaou NI, Arntz H-R, Bellou A (2015). European Resuscitation Council guidelines for resuscitation 2015 Section 8. Initial management of acute coronary syndromes. Resuscitation.

[11] Ong MEH, Wong ASL, Seet CM (2013). Nationwide improvement of door-to-balloon times in patients with acute st-segment elevation myocardial infarction requiring primary percutaneous coronary intervention with out-of-hospital 12-lead ecg recording and transmission. Ann Emerg Med.

[12] Pedersen SH, Galatius S, Hansen PR (2009). Field triage reduces treatment delay and improves long-term clinical outcome in patients with acute ST-segment elevation myocardial infarction treated with primary percutaneous coronary intervention. J Am Coll Cardiol.

[13] Perk J, De Backer G, Gohlke H (2012). European guidelines on cardiovascular disease prevention in clinical practice (Version 2012). Int J Behav Med.

[14] Quinn T, Johnsen S, Gale CP (2014). Effects of prehospital 12-lead ECG on processes of care and mortality in acute coronary syndrome: a linked cohort study from the Myocardial Ischaemia National Audit Project. Heart.

[15] Ravn-Fischer A, Karlsson T, Johanson P, Herlitz J (2013). Prehospital ECG signs of acute coronary occlusion are associated with reduced one-year mortality. Int J Cardiol.

[16] Roffi M, Patrono C, Collet J-P (2016). 2015 ESC Guidelines for the management of acute coronary syndromes in patients presenting without persistent ST-segment elevation. Eur Heart J.

[17] Statisches Bundesamt (2017). Gesundheit Todesursachen in Deutschland 2015.

[18] Tataris KL, Mercer MP, Govindarajan P (2015). Prehospital aspirin administration for acute coronary syndrome (ACS) in the USA: an EMS quality assessment using the NEMSIS 2011 database. Emerg Med J.

[19] Thang ND, Sundström BW, Karlsson T (2014). ECG signs of acute myocardial ischemia in the prehospital setting of a suspected acute coronary syndrome and its association with outcomes. Am J Emerg Med.

